# An External-Validated Prediction Model to Predict Lung Metastasis among Osteosarcoma: A Multicenter Analysis Based on Machine Learning

**DOI:** 10.1155/2022/2220527

**Published:** 2022-05-06

**Authors:** Wenle Li, Wencai Liu, Fida Hussain Memon, Bing Wang, Chan Xu, Shengtao Dong, Haosheng Wang, Zhaohui Hu, Xubin Quan, Yizhuo Deng, Qiang Liu, Shibin Su, Chengliang Yin

**Affiliations:** ^1^Department of Orthopedics, Xianyang Central Hospital, Xianyang, China; ^2^Clinical Medical Research Center, Xianyang Central Hospital, Xianyang, China; ^3^Department of Orthopaedic Surgery, The First Affiliated Hospital of Nanchang University, Nanchang, China; ^4^Department of Electrical Engineering, Sukkur IBA University, Pakistan; ^5^Department of Mechatronics Engineering, Jeju National University, Jeju, Republic of Korea; ^6^Department of Spine Surgery, Second Affiliated Hospital of Dalian Medical University, China; ^7^Department of Orthopaedics, The Second Hospital of Jilin University, Changchun, China; ^8^Department of Spine Surgery, Liuzhou People's Hospital, Liuzhou, China; ^9^Graduate School of Guangxi Medical University, Nanning, Guangxi, China; ^10^Study in School of Guilin Medical University, Guilin, Guangxi, China; ^11^Department of Business Management, Xiamen Bank, Xiamen, China; ^12^Faculty of Medicine, Macau University of Science and Technology, Macau, China

## Abstract

**Background:**

Lung metastasis greatly affects medical therapeutic strategies in osteosarcoma. This study aimed to develop and validate a clinical prediction model to predict the risk of lung metastasis among osteosarcoma patients based on machine learning (ML) algorithms.

**Methods:**

We retrospectively collected osteosarcoma patients from the Surveillance Epidemiology and End Results (SEER) database and from four hospitals in China. Six ML algorithms, including logistic regression (LR), gradient boosting machine (GBM), extreme gradient boosting (XGBoost), random forest (RF), decision tree (DT), and multilayer perceptron (MLP), were applied to build predictive models for predicting lung metastasis using patient's demographics, clinical characteristics, and therapeutic variables from the SEER database. The model was internally validated using 10-fold cross-validation to calculate the mean area under the curve (AUC) and the model was externally validated using the Chinese multicenter osteosarcoma data. Relative importance ranking of predictors was plotted to understand the importance of each predictor in different ML algorithms. The correlation heat map of predictors was plotted to understand the correlation of each predictor, selecting the 10-fold cross-validation with the highest AUC value in the external validation ROC curve to build a web calculator.

**Results:**

Of all enrolled patients from the SEER database, 17.73% (194/1094) developed lung metastasis. The multiple logistic regression analysis showed that sex, N stage, T stage, surgery, and bone metastasis were all independent risk factors for lung metastasis. In predicting lung metastasis, the mean AUCs of the six ML algorithms ranged from 0.711 to 0.738 in internal validation and 0.697 to 0.729 in external validation. Among the six ML algorithms, the extreme gradient boosting (XGBoost) model had the highest AUC value with an average internal AUC of 0.738 and an external AUC of 0.729. The best performing ML algorithm model was used to build a web calculator to facilitate clinicians to calculate the risk of lung metastasis for each patient.

**Conclusions:**

The XGBoost model may have the best prediction effect and the online calculator based on this model can help doctors to determine the lung metastasis risk of osteosarcoma patients and help to make individualized medical strategies.

## 1. Introduction

Osteosarcoma, the most common malignant bone tumor in children and adolescents, had an incidence of approximately 0.8–11/100,000 in people aged 15 to 19 years [1, 2]. Osteosarcoma usually occurs during a period of rapid bone growth . It is most commonly observed in the bones of the extremities [3], which is characterized by bone morbidity, such as pain and swelling . And it increases with age [1]. Patients who got metastatic osteosarcoma had a very poor prognosis, with only about 20% to 30% of them having long-term survival, while this proportion increased to 65% to 70% with nonmetastatic osteosarcoma [4, 5]. Of all metastatic sites, the most common site is the lung (85% to 90%), followed by bone metastases (8% to 10%) [6, 7]. Lung metastases contribute to the poor prognosis of most osteosarcoma patients, even after complete resection of the primary tumor. The treatment of patients with metastatic osteosarcoma remains controversial, and the majority of clinical trials excluded patients with metastatic osteosarcoma, resulting in inconsistent treatment modalities [3, 8, 9]. Thus, considering the dramatic impact of lung metastases on survival and treatment options for osteosarcoma patients, identifying osteosarcoma patients at a higher risk for lung metastases would have strong clinical implications.

Machine learning (ML), a form of artificial intelligence model, is widely used in healthcare data analysis [10–16]. By leveraging the powerful predictive capabilities of ML algorithms, clinical prediction models are superior to those developed by traditional statistical approaches [17–20]. Consequently, it is necessary to create new novel prediction models to better predict risk among osteosarcoma patients. With the clinical prediction models, clinicians are capable of assessing the risk of lung metastasis for each osteosarcoma patient and developing individual therapeutic strategies, such as adjuvant therapy and further optimizing treatment regimens [21]. However, there are no ML models to predict the risk of lung metastasis in osteosarcoma are available [22].

In this study, byusing patient's demographic, pathological, and clinical characteristics, we aimed to develop an ML-based model to predict the susceptibility of lung metastases in osteosarcoma patients. Then, the model was externally validated with data from four hospitals in China. Finally, the ML algorithm possessing the strongest predictive power was visualized and dynamized by a web-based calculator. As a tool for prediction, it could help doctors to determine the lung metastasis risk of osteosarcoma patients and make individualized medical strategies. It ultimately provided a basis for future treatment and prevention strategies.

## 2. Patients and Methods

### 2.1. Study Populations and Design

We retrospectively collected data from the SEER database and four hospitals in China including the Second Affiliated Hospital of Jilin University, the Second Affiliated Hospital of Dalian Medical University, Liuzhou People's Hospital, and Xianyang Central Hospital. Although the low incidence of osteosarcoma makes it very difficult to study large samples of patients, the Surveillance, Epidemiology, and End Results (SEER) database provides favorable resources for investigating rare malignancies in the settings where prospective data or clinical trials are limited. Thus, we used this common database to analyze rare cancers [23].

Patients with osteosarcoma diagnosed between 2010 and 2016 from the SEER database were used as the training cohort. The inclusion criteria were as follows: (1) diagnosis of ES with ICD-O-3/WHO 2008 morphology code 9180; (2) pathologically confirmed primary osteosarcoma; (3) absence of concurrent malignancies; (4) complete clinical data including age at diagnosis, race, survival time, tumor primary site, grade, bone metastasis, lung metastasis, laterality, T stage, N stage, surgery, radiotherapy, chemotherapy, and other demographic and clinical variables. Exclusion criteria were as follows: (1) missing or unavailable clinicopathological and survival time information; (2) cases with other primary tumor disease and metastatic status unknown; and (3) data gaps (blanks).

Osteosarcoma patients from the four medical institutions in China between 2010 and 2018 were used as the validation cohort. Patients were included if the diagnosis of osteosarcoma was pathologically confirmed and patients did not have other primary tumors. Patients were excluded if there were missing data or if the follow-up was less than two years. The follow-up deadline was December 1, 2020. At each institution, patients were followed up for at least two years and clear clinical pathological and follow-up information was recorded. Information retrieved included patient demographics (race, gender, and age at diagnosis), tumor characteristics (primary site, grade, laterality, T stage, N stage, lung metastases, and bone metastases), and follow-up data for treatment (surgery, chemotherapy, and radiation therapy).

### 2.2. Definition of Predictive Variables

All potential predictors were standardized in the study. There were three categories of race in the SEER data, white, black, and other, and other did not have a specific ethnicity. So the multicenter data from China were all classified as other. Treatment modalities included surgery, chemotherapy, and radiotherapy, and they were categorized as “No” or “Yes.” All potential predictors included race (black vs. other vs. white), age (median [interquartile range (IQR)]), sex (female vs. male), primary site (axis bone vs. limb bone vs. other), grade (moderately differentiated vs. poorly differentiated vs. undifferentiated; anaplastic vs. unknown vs. well differentiated), laterality (left vs. not a paired site vs. right), T (T1 vs. T2 vs. T3 vs. TX), N (N0 vs. N1 vs. NX), surgery (No vs. Yes), radiation (No vs. Yes), chemotherapy (No vs. Yes), bone metastases (No vs. Yes), lung metastases (No vs. Yes), and survival time (median [IQR]). T indicates primary tumor, TX means the primary tumor is unknown, T0 represents no evidence of primary tumor, T1 means tumors are confined to the bone cortex, and T2 means tumor exceeds the bone cortex. N is regional lymph node metastasis: Lymph nodes in NX area are unknown, N0 tumors have no regional lymph node metastasis, and N1 tumors have regional lymph node metastasis. Survival time was defined as the time interval between the surgery date and death date.

### 2.3. Development and Validation of Prediction Models

ML algorithms outperform traditional regression methods when it comes to predicting outcomes [12, 18, 24–26]. This study used six machine learning algorithms to build the models: logistic regression (LR), gradient boosting machine (GBM), extreme gradient boosting (XGBoost), random forest (RF), decision tree (DT), and multilayer perceptron (MLP). XGBoost is an integration algorithm based on boost. It is typical of the integration of cart tree, which is an improvement of the gradient tree boosting. During training, the training cohort internal validation method uses 10-fold cross-validation to evaluate the predictive power of each machine learning classifier in plotting the average AUC.

Using the validation cohort, six machine learning models ROCwere plotted and AUCswere calculated to evaluate the predictive ability of the models in different cohorts. In the performance comparison of machine learning algorithms, the AUC iscloser to 1, the better the classification model . Subsequently, based on the best predictive ability model, we created an online risk calculator that can make predictions using newly entered data of patients with osteosarcoma, thus enabling clinicians to easily and more accurately predict the risk of lung metastasis in these patients. Using the permutation importance principle, the results of 100 independent training simulations were created to assess the importance of the predictors for each ML model predicting lung metastasis. A correlation heat map of the predictors was created to assess the correlation of each predictor.

### 2.4. Statistical Analysis

We extracted data from the SEER database using SEER *∗* STAT (8.3.5) software. Baseline characteristics of the training cohort and validation cohort were compared using chi-square tests and independent samples *t*-tests. Univariate logistic regression analysis was performed to assess risk factors predicting lung metastasis in the training cohort of patients with osteosarcoma. Predictors with *P* < 0.05 in the results of the univariate logistic were included in the multivariate logistics regression analysis. Results with *P* < 0.05 as an independent risk factor were included in the predictive model of the machine learning algorithm. A backward stepwise selection method was used to calculate the dominance ratio (OR) with a confidence interval (CI) of 95%. Statistical analyses were performed using R software (version 4.1.1). Machine learning models and web calculators were built using Python (version 3.8). *P* < 0.05 was considered statistically significant.

## 3. Results

### 3.1. Baseline Patient Characteristics

After inclusion and exclusion, a total of 1201 patients were included. There were significant differences between the training and validation groups in terms of race and duration of radiotherapy (*P* < 0.05). The ethnic composition of the patients from the Chinese multicenter was Chinese, which was categorized as “other” in the SEER database. Also, a higher proportion of patients from China were treated with chemotherapy. The remaining parameters: lung metastasis, age, survival time, gender, site of origin, grade, T stage, N stage, surgery, radiotherapy, and bone metastasis, were not statistically significant (Table 1). Notably, of all enrolled patients from SEER database, 17.73% (194/1094) developed lung metastasis. Among all patients from the multicenter analysis, 18.69% (20/107) had lung metastasis.

There were statistically significant differences in gender, T stage, N stage, surgery, radiotherapy, bone metastases, and survival time between patients with and without lung metastases at baseline, with no statistical differences in the remaining variables (Table 2). In detail, patients with lung metastases had a higher proportion of males, higher T stage and N stage grades, higher use of radiotherapy and bone metastases, and shorter survival time (Table 2).

### 3.2. Univariate and Multivariate Logistic Regression

Univariate logistic regression analysis identified six risk factors associated with lung metastases, including gender, N stage, T stage, surgery, radiotherapy, and bone metastases (Table 3). According to the multivariate logistic regression analysis, the results showed that gender, N stage, T stage, surgery, and bone metastasis were independent risk factors for lung metastasis. Among them, the female was an independent protective factor for lung metastasis, and T stage (T2, T3, TX), N stage (N1, NX), failure to undergo surgery, and bone metastasis were independent risk factors for lung metastasis.

### 3.3. Performance of the Machine Learning Algorithm

**Figure 1:**
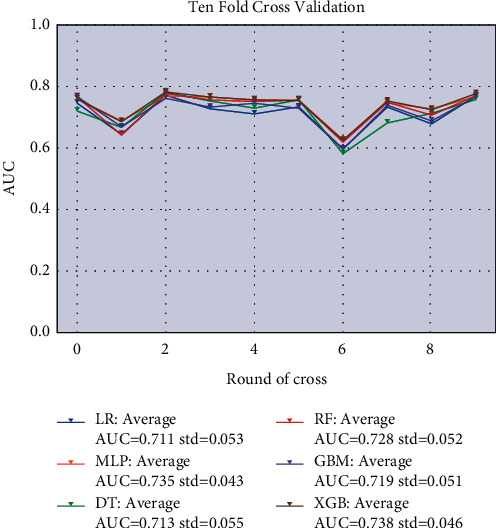
10-fold cross-validation of machine learning algorithms.

**Figure 2:**
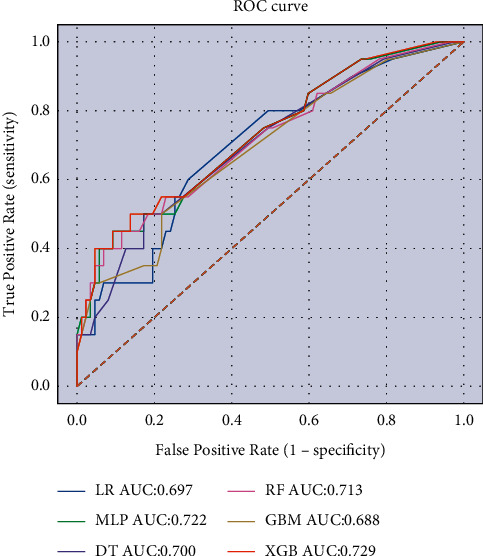
ROC curves of six ML algorithm models in predicting the risk of lung metastasis in osteosarcoma patients.

The machine learning algorithm's performance was validated in the training set with 10-fold cross-validation, and the results were shown in Figure 1. It showed that the XGBoost model exhibited the highest performance in predicting lung metastasis with a p-average of 0.738. The external validation results of the model using the validation set were shown in Figure 2, which showed that the XGBoost model still showed the highest performance in predicting lung metastasis in the external data cohort with AUC = 0.729. Therefore, we chose the XGBoost model as the final prediction model.

### 3.4. Relative Importance and Correlation of Variables

**Figure 3:**
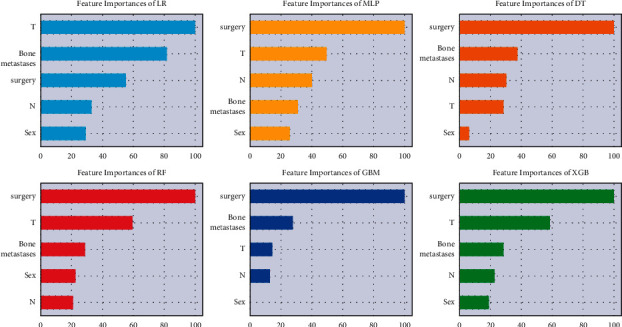
Relative importance ranking of features in ML algorithms for predicting lung metastasis.

**Figure 4:**
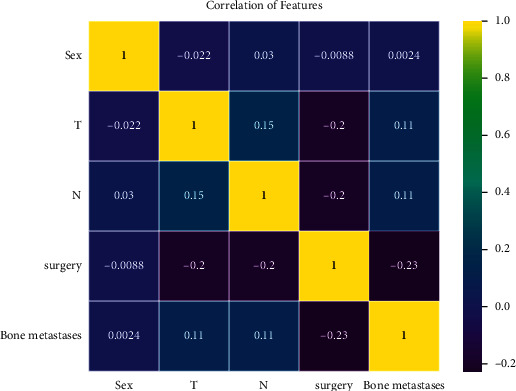
The correlation of variables. Yellow indicates positive correlation and purple indicates negative correlation.

Figure 3 showed the relative importance of variables in each of the lung metastasis prediction ML algorithms. We could observe a trend in the prediction variables: although the importance of the variables varied slightly among the different ML algorithms, surgery was in the first place in five algorithms, and T stage and bone metastasis were also in the top three among the five algorithms. In contrast, sex was the last among the five algorithms. The importance of the high-level variables in the XGBoost model was ranked in descending order as follows: surgery, T stage, bone metastases, N stage, and sex.  Figure 4 showed the correlation of variables in the lung metastasis prediction ML algorithms. We could observe that there was no clear positive correlation for all variables. Surgery had a significant negative correlation with three variables: T stage, N stage, and bone metastases.

### 3.5. Web-Based Calculator

**Figure 5:**
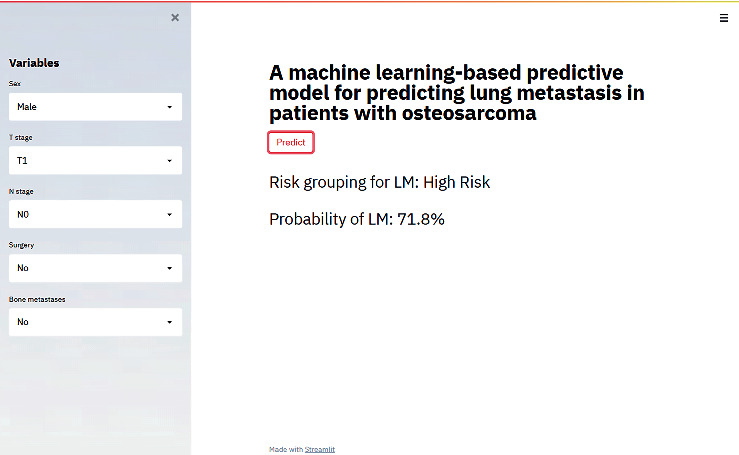
The web calculator predicting lung metastases in patients with osteosarcoma.

A web-based calculator was built based on the most predictive XGBoost algorithm for clinicians to predict the risk of lung metastasis in osteosarcoma patients (https://share.streamlit.io/liuwencai123/os_lm/main/os_lm.py) (Figure 5). This calculator was easy to use and doctors could calculate the probability of developing lung metastasis for each osteosarcoma patients simply by entering easily available preoperative and intraoperative clinicopathological variables. The probability would automatically present by clicking the “predict” button.

## 4. Discussion

Metastasis from sarcoma is confined to the lung, and metastasectomy is an important component of the management of sarcoma. A study found that 81% had lung metastases and 62% had only lung metastases among 202 sarcoma patients [8]. This study developed and validated several machine learning algorithms to predict lung metastasis in osteosarcoma patients. The results showed that the XGBoost model had the best predictive power in both internal and external validation. To make the clinical application of this model feasible, we built a web calculator to visualize the model for estimating the individual probability of lung metastasis in each osteosarcoma patient. This ML-based model can guide clinicians to target each patient's treatment plan, making precision medicine possible.

The proportion of male patients was slightly higher than that of female in both the US SEER data and the Chinese multicenter cohort. The results of the logistics analysis showed that the risk of lung metastasis was 0.58 times lower in female patients than in male patients. To our knowledge, this was the first study to focus on the effect of gender on lung metastasis from osteosarcoma. One study found that the mean age of the onset of osteosarcoma was 10 to 14 years for women and 15 to 19 years for men. We, therefore, speculated that differences in sex hormone levels during the development of secondary sexual characteristics might contribute to the differences in tumor aggressiveness.

The multiple logistic regression analysis showed that the risk of lung metastasis was much higher in T2, T3, and TX than in T1, and the risk increased with a larger volume. Previous studies have shown that patients with smaller osteosarcoma had better survival expectations. A larger tumor volume means that the tumor had a longer growth cycle, and the tumor was more aggressive and invasive and was therefore prone to lung metastases. Tumor size also influenced treatment strategies, with a correlation heat map showing a negative correlation between T and surgery. Larger tumor volumes were challenging for the surgeon since the likelihood of complete resection of the tumor was declining. In N stage, patients with NX stage were significantly more likely to develop lung metastasis than patients with other stages. The proportion of patients with definite lymphatic metastases (N1) was low in both the training and validation cohorts, neither exceeding 5%. However, the proportion of N1 versus NX was higher in patients presenting with lung metastases than in the nonmetastatic group. Therefore, we believed that having lymphatic metastases indicated that the osteosarcoma was very aggressive. One study found that patients presenting with lymphatic local metastases or distal metastases had significantly lower survival rates than other patients [24, 25]. The majority of osteosarcoma patients who suffered from mortality were mainly due to lung metastases. Osteosarcoma was relatively rare in general nonspecialized bone oncology specialties, and osteosarcoma presenting with lymphatic metastases was even rarer. Considering the correlation of lymphatic metastasis with lung metastasis, examination of lymphatic metastasis could not be ignored by clinicians.

Bone metastases were also not common among osteosarcoma and did not exceed 5% in either cohort. However, of the 55 patients who presented with bone metastases in this study, 35 had concomitant lung metastases. Bone metastases were a manifestation of multimetastatic disease, and patients presenting with isolated bone metastases at presentation were rare. Thus, we recommended that patients with bone metastases or multifocal osteosarcoma should be further examined for lung metastases. Patients without lung metastases underwent surgery in 85.5%, much higher than the 61.7% of patients with lung metastases. When the tumor was considered unresectable or difficult to resect, namely, when the T or N stage was advanced, chemotherapy or radiation therapy was first recommended, followed by periodic reassessment of tumor resectability. Regarding some cases, surgical resection was extremely challenging for the surgeon, but every effort should still be made to pursue surgical opportunities [27–29].

To our knowledge, this study was the first study of attempting to predict osteosarcoma lung metastasis using machine learning algorithms. Besides, this study was also the first multicenter osteosarcoma study to use both the US SEER database and data from multiple medical centers in China. Some previous prediction models for osteosarcoma based on the SEER database have developed based on the SEER database alone, and it was not clear whether they could be used in different regions [30–32]. Also, all of these studies used only the nomogram as a visual prediction model and did not provide a dynamic prediction model, and they had some drawbacks in terms of convenience. More importantly, most studies on prediction models for osteosarcoma patients were single-center studies without external validation in different patient cohorts, and validity, clinical utility were greatly compromised [33–35]. Therefore, we collected data on osteosarcoma patients from four medical centers in different regions of China as a validation group to validate the model's predictive power and its value for use in different regions. Furthermore, we built a web calculator based on the XGBoost algorithm model, which had the best ability to predict the risk of lung metastasis to increase the clinical utility of the model. Clinicians were capable of calculating the risk of lung metastasis for each patient with osteosarcoma and thus personalizing their treatment plans.

However, despite our best efforts to improve it, this study still had limitations. First, retrospective studies might lead to data bias. Second, although we externally validated the model using different patient cohorts, prospective studies were needed to determine whether it improved patient outcomes. Third, the information currently available in SEER's clinical database was somewhat limited, and many more details such as specific protocols for surgical margins and radiotherapy were not available, which would further improve the predictive power of the model if these data were included in the model.

## 5. Conclusions

Through multiple logistic regression analysis, we have showed that sex, N stage, T stage, surgery, and bone metastasis were all independent risk factors for lung metastasis. The mean AUCs of the six ML algorithms ranged from 0.711 to 0.738 in internal validation and 0.697 to 0.729 in external validation. Among the six ML algorithms, the XGBoost showed the best performance with an average internal AUC of 0.738 and an external AUC of 0.729.

The XGBoost model may have the best prediction effect and the online calculator based on this model can help doctors to determine the lung metastasis risk of osteosarcoma patients and help to make individualized medical strategies.

## Figures and Tables

**Table 1:** Baseline data table of the training group and the validation group.

	Level	Overall (*N* = 1201)	Multicenter (validation group, *N* = 107)	SEER (training group, *N* = 1094)	*P*

Race (%)	Black	163 (13.57)	0 (0.00)	163 (14.90)	<0.0001
	Other	216 (17.99)	107 (100.00)	109 (9.96)	
	White	822 (68.44)	0 (0.00)	822 (75.14)	

Age (median [IQR])	NA	21.000 [13.000, 53.000]	18.000 [13.000, 48.500]	22.000 [13.000, 53.750]	0.3895

Sex (%)	Female	549 (45.71)	50 (46.73)	499 (45.61)	0.9048
	Male	652 (54.29)	57 (53.27)	595 (54.39)	

Primary site (%)	Axis bone	313 (26.06)	25 (23.36)	288 (26.33)	0.374
	Limb bone	787 (65.53)	76 (71.03)	711 (64.99)	
	Other	101 (8.41)	6 (5.61)	95 (8.68)	

Grade (%)	Moderately differentiated	41 (3.41)	0 (0.00)	41 (3.75)	0.0934
	Poorly differentiated	294 (24.48)	22 (20.56)	272 (24.86)	
	Undifferentiated; anaplastic	553 (46.04)	49 (45.79)	504 (46.07)	
	Unknown	286 (23.81)	34 (31.78)	252 (23.03)	
	Well differentiated	27 (2.25)	2 (1.87)	25 (2.29)	

Laterality (%)	Left	514 (42.80)	40 (37.38)	474 (43.33)	0.0524
	Not a paired site	161 (13.41)	9 (8.41)	152 (13.89)	
	Right	526 (43.80)	58 (54.21)	468 (42.78)	

T (%)	T1	420 (34.97)	38 (35.51)	382 (34.92)	0.1914
	T2	562 (46.79)	44 (41.12)	518 (47.35)	
	T3	40 (3.33)	7 (6.54)	33 (3.02)	
	TX	179 (14.90)	18 (16.82)	161 (14.72)	

N (%)	N0	1088 (90.59)	91 (85.05)	997 (91.13)	0.12
	N1	36 (3.00)	5 (4.67)	31 (2.83)	
	NX	77 (6.41)	11 (10.28)	66 (6.03)	

Surgery (%)	No	225 (18.73)	23 (21.50)	202 (18.46)	0.5241
	Yes	976 (81.27)	84 (78.50)	892 (81.54)	

Radiation (%)	No	1054 (87.76)	100 (93.46)	954 (87.20)	0.0837
	Yes	147 (12.24)	7 (6.54)	140 (12.80)	

Chemotherapy (%)	No	236 (19.65)	10 (9.35)	226 (20.66)	0.0073
	Yes	965 (80.35)	97 (90.65)	868 (79.34)	

Bone metastases (%)	No	1144 (95.25)	102 (95.33)	1042 (95.25)	1
	Yes	57 (4.75)	5 (4.67)	52 (4.75)	

Lung metastases (%)	No	987 (82.18)	87 (81.31)	900 (82.27)	0.9085
	Yes	214 (17.82)	20 (18.69)	194 (17.73)	

Times (median [IQR])	NA	24.000 [12.000, 48.000]	23.000 [11.000, 51.500]	24.000 [12.000, 47.000]	0.9754

Abbreviation: SEER, Surveillance Epidemiology and End Results; IQR, interquartile range; T, tumor; N, lymph node.

**Table 2:** Baseline data for patients presenting with and without lung metastases.

	Level	Overall (*N* = 1201)	No (*N* = 987)	Yes (*N* = 214)	*P*
Race (%)	Black	163 (13.6)	132 (13.4)	31 (14.5)	0.847
	Other	216 (18.0)	176 (17.8)	40 (18.7)	
	White	822 (68.4)	679 (68.8)	143 (66.8)	
Age (mean (SD))	NA	32.98 (24.08)	32.83 (23.67)	33.69 (25.93)	0.637

Sex (%)	Female	549 (45.7)	471 (47.7)	78 (36.4)	0.003
	Male	652 (54.3)	516 (52.3)	136 (63.6)	

Primary site (%)	Axis bone	313 (26.1)	263 (26.6)	50 (23.4)	0.468
	Limb bone	787 (65.5)	639 (64.7)	148 (69.2)	
	Other	101 (8.4)	85 (8.6)	16 (7.5)	

Grade (%)	Moderately differentiated	41 (3.4)	36 (3.6)	5 (2.3)	0.206
	Poorly differentiated	294 (24.5)	236 (23.9)	58 (27.1)	
	Undifferentiated; anaplastic	553 (46.0)	450 (45.6)	103 (48.1)	
	Unknown	286 (23.8)	239 (24.2)	47 (22.0)	
	Well differentiated	27 (2.2)	26 (2.6)	1 (0.5)	

Laterality (%)	Left	514 (42.8)	425 (43.1)	89 (41.6)	0.426
	Not a paired site	161 (13.4)	137 (13.9)	24 (11.2)	
	Right	526 (43.8)	425 (43.1)	101 (47.2)	

Stage group (%)	I	198 (16.5)	194 (19.7)	4 (1.9)	<0.001
	II	562 (46.8)	550 (55.7)	12 (5.6)	
	III	51 (4.2)	50 (5.1)	1 (0.5)	
	IV	278 (23.1)	83 (8.4)	195 (91.1)	
	UNK stage	112 (9.3)	110 (11.1)	2 (0.9)	

T (%)	T1	420 (35.0)	382 (38.7)	38 (17.8)	<0.001
	T2	562 (46.8)	448 (45.4)	114 (53.3)	
	T3	40 (3.3)	25 (2.5)	15 (7.0)	
	TX	179 (14.9)	132 (13.4)	47 (22.0)	

N (%)	N0	1088 (90.6)	913 (92.5)	175 (81.8)	<0.001
	N1	36 (3.0)	24 (2.4)	12 (5.6)	
	NX	77 (6.4)	50 (5.1)	27 (12.6)	

M (%)	M0	931 (77.5)	911 (92.3)	20 (9.3)	<0.001
	M1	270 (22.5)	76 (7.7)	194 (90.7)	

Surgery (%)	No	225 (18.7)	143 (14.5)	82 (38.3)	<0.001
	Yes	976 (81.3)	844 (85.5)	132 (61.7)	

Radiation (%)	No	1054 (87.8)	878 (89.0)	176 (82.2)	0.009
	Yes	147 (12.2)	109 (11.0)	38 (17.8)	

Chemotherapy (%)	No	236 (19.7)	204 (20.7)	32 (15.0)	0.07
	Yes	965 (80.3)	783 (79.3)	182 (85.0)	

Bone metastases (%)	No	1144 (95.3)	965 (97.8)	179 (83.6)	<0.001
	Yes	57 (4.7)	22 (2.2)	35 (16.4)	

Category (%)	Multicenter data (validation group)	107 (8.9)	87 (8.8)	20 (9.3)	0.908
	SEER data (training group)	1094 (91.1)	900 (91.2)	194 (90.7)	
Times (mean (SD))	NA	30.32 (22.75)	32.96 (22.96)	18.13 (17.12)	<0.001

**Table 3:** Univariate and multivariate logistic regression analysis of risk factors for lung metastasis in patients with osteosarcoma.

Variables	Univariate OR (95% CI)	*P* value	Multivariate OR (95% CI)	*P* value
Age (years)	1.001 (0.995–1.008)	0.637	—	—
Race				
White	Ref	Ref	Ref	Ref
Black	1.115 (0.725–1.716)	0.620	—	—
Other	1.079 (0.732–1.590)	0.700	—	—
Sex				
Male	Ref	Ref	Ref	Ref
Female	0.628 (0.463–0.853)	<0.05	0.586 (0.419–0.819)	<0.05
Primary site				
Limb bones	Ref	Ref	Ref	Ref
Axis of a bone	0.821 (0.578–1.166)	0.271	—	—
Other	0.813 (0.463–1.427)	0.471	—	—
Grade				
Well differentiated	Ref	Ref	Ref	Ref
Moderately differentiated	3.611 (0.398–32.770)	0.254	—	—
Poorly differentiated	6.390 (0.849–48.065)	0.072	—	—
Undifferentiated; anaplastic	5.951 (0.798–44.359)	0.082	—	—
Unknown	5.113 (0.677–38.606)	0.114	—	—
Laterality				
Left	Ref	Ref	Ref	Ref
Right	1.135 (0.828–1.555)	0.431	—	—
Other	0.837 (0.512–1.366)	0.475	—	—
T				
T1	Ref	Ref	Ref	Ref
T2	2.558 (1.729–3.785)	<0.001	2.331 (1.542–3.524)	<0.001
T3	6.032 (2.931–12.413)	<0.001	4.154 (1.834–9.407)	<0.01
TX	3.579 (2.235–5.734)	<0.001	2.067 (1.205–3.545)	<0.01
N				
N0	Ref	Ref	Ref	Ref
N1	2.609 (1.280–5.314)	<0.01	1.315 (0.572–3.023)	0.519
NX	2.817 (1.717–4.623)	<0.001	2.040 (1.143–3.640)	<0.05
Surgery				
No	Ref	Ref	Ref	Ref
Yes	0.273 (0.197–0.378)	<0.001	0.574 (0.383–0.859)	<0.01
Radiation				
No	Ref	Ref	Ref	Ref
Yes	1.739 (1.162–2.603)	<0.05	1.244 (0.781–1.979)	0.358
Chemotherapy				
No	Ref	Ref	Ref	Ref
Yes	1.482 (0.987–2.224)	0.058	—	—
Bone metastases				
No	Ref	Ref	Ref	Ref
Yes	8.577 (4.916–14.964)	<0.001	4.542 (2.451–8.414)	<0.001

## Data Availability

The data that support the findings of this study are available from SEER registry but restrictions apply to the availability of these data, which were used under license for the current study, and so are not publicly available. All multicenter data generated or analyzed during this study are included in this published article.

## References

[1] Casali P. G., Bielack S., Abecassis N. (2018). Bone sarcomas: ESMO-PaedCan-EURACAN Clinical Practice Guidelines for diagnosis, treatment and follow-up. *Annals of Oncology*.

[2] Valery P. C., Laversanne M., Bray F. (2015). Bone cancer incidence by morphological subtype: a global assessment. *Cancer Causes & Control*.

[3] Meazza C., Scanagatta P. (2016). Metastatic osteosarcoma: a challenging multidisciplinary treatment. *Expert Review of Anticancer Therapy*.

[4] Ferrari S., Palmerini E. (2007). Adjuvant and neoadjuvant combination chemotherapy for osteogenic sarcoma. *Current Opinion in Oncology*.

[5] Grünewald T. G., Alonso M., Avnet S. (2020). Sarcoma treatment in the era of molecular medicine. *EMBO Molecular Medicine*.

[6] Chou A. J., Geller D. S., Gorlick R. (2008). Therapy for osteosarcoma. *Pediatric Drugs*.

[7] Liang S., Ren Z., Han X. (2015). PLA2G16 expression in human osteosarcoma is associated with pulmonary metastasis and poor prognosis. *PLoS One*.

[8] Treasure T., Macbeth F. (2016). Is surgery warranted for oligometastatic disease?. *Thoracic Surgery Clinics*.

[9] Treasure T., Milošević M., Fiorentino F., Macbeth F. (2014). Pulmonary metastasectomy: what is the practice and where is the evidence for effectiveness?: table 1. *Thorax*.

[10] Wang H., Tang Z.-R., Li W. (2021). Prediction of the risk of C5 palsy after posterior laminectomy and fusion with cervical myelopathy using a support vector machine: an analysis of 184 consecutive patients. *Journal of Orthopaedic Surgery and Research*.

[11] Wang H., Fan T., Yang B., Lin Q., Li W., Yang M. (2021). Development and internal validation of supervised machine learning algorithms for predicting the risk of surgical site infection following minimally invasive transforaminal lumbar interbody fusion. *Frontiers of Medicine*.

[12] Li W., Wang J., Liu W. (2021). Machine learning applications for the prediction of bone cement leakage in percutaneous vertebroplasty. *Frontiers in Public Health*.

[13] Wu E. Q., Hu D., Deng P.-Y. (2021). Nonparametric bayesian prior inducing deep network for automatic detection of cognitive status. *IEEE Transactions on Cybernetics*.

[14] Wu E. Q., Lin C.-T., Zhu L.-M., Tang Z. R., Jie Y.-W., Zhou G.-R. (2021). Fatigue detection of pilots’ brain through brains cognitive map and multilayer latent incremental learning model. *IEEE Transactions on Cybernetics*.

[15] Tang Z., Chen Y., Ye S. (2020). Fully memristive spiking-neuron learning framework and its applications on pattern recognition and edge detection. *Neurocomputing*.

[16] Tang Z., Zhu R., Hu R. (2020). A multilayer neural network merging image preprocessing and pattern recognition by integrating diffusion and drift memristors. *IEEE Transactions on Cognitive and Developmental Systems*.

[17] Deo R. C. (2015). Machine learning in medicine. *Circulation*.

[18] Tang Z., Zhu R., Lin P. (2019). A hardware friendly unsupervised memristive neural network with weight sharing mechanism. *Neurocomputing*.

[19] Li W., Wang H., Dong S. (2021). Establishment and validation of a nomogram and web calculator for the risk of new vertebral compression fractures and cement leakage after percutaneous vertebroplasty in patients with osteoporotic vertebral compression fractures. *European Spine Journal*.

[20] Li W., Dong S., Wang B. (2022). The construction and development of a clinical prediction model to assess lymph node metastases in osteosarcoma. *Frontiers in Public Health*.

[21] Khoo C. Y., Chai X., Quek R., Teo M. C. C., Goh B. K. P. (2018). Systematic review of current prognostication systems for primary gastrointestinal stromal tumors. *European Journal of Surgical Oncology*.

[22] Li W., Dong S., Wang H. (2021). Risk analysis of pulmonary metastasis of chondrosarcoma by establishing and validating a new clinical prediction model: a clinical study based on SEER database. *BMC Musculoskeletal Disorders*.

[23] Doll K. M., Rademaker A., Sosa J. A. (2018). Practical guide to surgical data sets: surveillance, Epidemiology, and End results (SEER) database. *JAMA surgery*.

[24] Miller B. J., Cram P., Lynch C. F., Buckwalter J. A. (2013). Risk factors for metastatic disease at presentation with osteosarcoma. *Journal of Bone and Joint Surgery*.

[25] Bacci G., Ferrari S., Longhi A. (2002). High-grade osteosarcoma of the extremity: differences between localized and metastatic tumors at presentation. *Journal of pediatric hematology/oncology*.

[26] Wu E. Q., Deng P.-Y., Qu X.-Y., Tang Z., Zhang W.-M., Zhu L.-M. (2020). *Detecting Fatigue Status of Pilots Based on Deep Learning Network Using EEG Signals*.

[27] Pastorino U. (2002). History of the surgical management of pulmonary metastases and development of the International Registry. *Seminars in Thoracic and Cardiovascular Surgery*.

[28] Huang Y.-M., Hou C.-H., Hou S.-M., Yang R.-S. (2009). The metastasectomy and timing of pulmonary metastases on the outcome of osteosarcoma patients. *Clinical Medicine. Oncology*.

[29] Pfannschmidt J., Egerer G., Bischof M., Thomas M., Dienemann H. (2012). Surgical intervention for pulmonary metastases. *Deutsches Ärzteblatt international*.

[30] Zhang J., Yang J., Wang H.-Q. (2019). Development and validation of a nomogram for osteosarcoma-specific survival. *Medicine*.

[31] Yang Q.-K., Lai Q.-Y., Wang Y., Wang Y., Yao Z.-X., Zhang X.-J. (2021). Establishment and validation of prognostic nomograms to predict overall survival and cancer-specific survival for patients with osteosarcoma. *Neoplasma*.

[32] Lu S., Wang Y., Liu G. (2021). Construction and validation of nomogram to predict distant metastasis in osteosarcoma: a retrospective study. *Journal of Orthopaedic Surgery and Research*.

[33] He Y., Liu H., Wang S., Zhang J. (2020). A nomogram for predicting cancer-specific survival in patients with osteosarcoma as secondary malignancy. *Scientific Reports*.

[34] Fu P., Shi Y., Chen G., Fan Y., Gu Y., Gao Z. (2020). Prognostic factors in patients with osteosarcoma with the surveillance, Epidemiology, and End results database. *Technology in Cancer Research and Treatment*.

[35] Wu E. Q., Hu D., Deng P.-Y., Tang Z., Cao Y., Zhang W.-M. (2020). Nonparametric bayesian prior inducing deep network for automatic detection of cognitive status. *IEEE Transactions on Cybernetics*.

